# Effects of Vestibular Rehabilitation in the Management of a Vestibular Migraine: A Review

**DOI:** 10.3389/fneur.2018.00440

**Published:** 2018-06-12

**Authors:** Ahmad H. Alghadir, Shahnawaz Anwer

**Affiliations:** Rehabilitation Research Chair, College of Applied Medical Sciences, King Saud University, Riyadh, Saudi Arabia

**Keywords:** dizziness, migraine, physical therapy, vestibular rehabilitation, vertigo

## Abstract

Vestibular rehabilitation (VR) has been shown to be effective for many vestibular disorders. This review focuses on the current evidence on the effects of physical therapy in the management of vestibular symptoms in individuals with a vestibular migraine (VM). The individuals with a history of a migraine tend to have a high incidence of vestibular symptoms with some or all of their headaches. A total of six included studies investigated the effects of VR in the management of VM. The critical review form for quantitative studies was used to appraise quality assessment and risk of bias in the selected studies. Previous studies validated the use of VR in the treatment of vestibular symptoms for individuals with a VM to include improved headache and migraine-related disability in patients with a VM. From the current evidence, it is difficult to provide conclusive evidence regarding the efficacy of VR to minimize vestibular symptoms in patients with VM. Therefore, more randomized controlled studies are required to make firm evidence on the effect of VR in reducing vestibular symptoms in patients with VM. The future prospective, blinded, randomized controlled studies may help to isolate possible therapeutic effects of VR and other general effects.

## Introduction

Migraine is the most prevalent neurological disorder affecting about 1 out of every 7 Americans annually, and results in numerous emergency department and outpatient visits (1). Migraine is considered an important public health problem, especially among women during their reproductive years (1). A migraine is a primary headache disorder characterized by recurrent headaches that are moderate to severe (2). The component of a migraine headache may be present with associated neuro-otological symptoms such as dizziness, vertigo, and unsteadiness (3–5). When the vestibular symptoms are attributed to a migraine mechanism, it is currently known as a vestibular migraine (VM). Migraine-related vestibular disorders may be developed by increased excitability of sensory information processing. Recently, the association between a migraine and vestibular symptoms has been demonstrated by a larger population-based study indicating that people with VM are more susceptible to have vestibular symptoms such as vertigo (6). VM is a frequent cause of intermittent vertigo in adults. The patients may have complained of dizziness, vertigo or imbalance (7–9). In very severe vestibular symptoms in migraine context, the individuals may have manifestations of nausea, vomiting (10), motion sensitivity (visual vertigo) (8, 11–13), and postural instability (4, 13). The most current findings on migraine treatment have shown a limited effectiveness at reducing or preventing acute attacks of vertigo (14). However, the access of drug therapy has been shown to be as low as 36%, which further reduced to 13% at follow-up even though most of the patients had ongoing vertigo symptoms (15).

Vestibular rehabilitation (VR) is a therapeutic approach to treat dizziness and balance dysfunction, and is based on central mechanisms of neuroplasticity, which includes adaptation, habituation, and substitution, that facilitate vestibular compensation (16, 17). VR has been shown to be effective for many vestibular disorders (8, 18–25). Herdman et al. (26) reported improvement in postural stability after VR in patients with post acoustic neuroma resection. Similarly, the patients of vestibular neurectomy, labyrinthectomy, and Meniere's disease were shown successful outcomes after VR (23). This article briefly reviews the prevalence, diagnosis, clinical manifestation, and treatment of a VM and focuses the role of VR in the management of vestibular symptoms in individuals with a VM.

## Epidemiology of the association of vestibular migraine and dizziness

The current scientific definitions of vertigo, imbalance, and dizziness are based on the recommendations of the International Bárány Society for Neuro-Otology. Vertigo is the self-perceived feeling of motion when no self-motion is happening; imbalance or unsteadiness is a self-perceived feeling about instability while sitting, standing, or walking without a specific directional choice; and dizziness is a self-perceived feeling of altered or impaired spatial orientation without a faulty or distorted sense of motion (27). All three of these can occur in peripheral, central and higher vestibular disorders (28).

The prevalence of a VM is more common as compared to other vestibular disorders such as Meniere's disease and vestibular neuritis (29, 30), particularly in women (women to men ratio is 5:1) (31). The annual prevalence of a VM is 0.89%, as reported in previously published studies (3, 32). A recent study confirms that VM is the most commonly seen conditions in the balance disorders clinic, with 41% of patients reported in the clinic were diagnosed with VM (33).

Previous epidemiological studies reported consistent evidence for an association between a migraine and vestibular symptoms. von Brevern and Neuhauser reported a significantly higher prevalence of migraine patients in the dizzy clinic as compared to the orthopedic clinic (34). In one study, over 50% headache sufferers reported dizziness or vertigo with their headaches (35). Vukovic et al. (36) reported a more frequent history of vertigo in migraine patients compared to headache-free controls.

## Diagnostic criteria of VM

The diagnosis of a VM for a patient presents an unnerving challenge to the physician for various reasons (18). The absence of any pathognomonic signs, biomarkers, blood, or lab tests and the presence of overlap symptoms between the VM and the other causes of vertigo make it more difficult to confirm the diagnosis of VM. In addition, the “International Headache Society (IHS)” is given diagnostic criteria for a basilar migraine that includes “Vertigo” as a symptom (37). Hence, to diagnose VM, one must exclude all other causes of vertigo.

Neuhauser et al. (31) suggested a set of guidelines which are sub-divided into obvious and apparent VM criteria based on migraine diagnosis given by IHS and eliminating all other probable causes by the pertinent Neuro-otological investigation. Recently, Lempert et al. (38) published a consensus document of the “Barany Society and the International Headache Society” for the diagnostic criteria of VM (Table 1).

**Table 1:** Diagnostic criteria for vestibular migraine (38).

	**Vestibular migraine**
A.	At least 5 episodes with vestibular symptoms of moderate or severe intensity, lasting 5 min to 72 h.
B.	Current or previous history of migraine with or without aura according to the International Classification of Headache Disorders (ICHD).
C.	One or more migraine features with at least 50% of the vestibular episodes: – headache with at least two of the following characteristics: one sided location, pulsating quality, moderate or severe pain intensity, aggravation by routine physical activity – photophobia and phonophobia, – visual aura.
D.	Not better accounted for by another vestibular or ICHD diagnosis.
	**Probable vestibular migraine**
A.	At least 5 episodes with vestibular symptoms of moderate or severe intensity, lasting 5 min to 72 h.
B.	Only one of the criteria B and C for vestibular migraine is fulfilled (migraine history or migraine features during the episode).
C.	Not better accounted for by another vestibular or ICHD diagnosis.

## Clinical manifestations of VM

The clinical manifestation of VM varies for each patient, including frequency and duration of episodes (30, 31). The history of spontaneous or positional vertigo is the typical feature of VM (31). Some patients have reported positional vertigo after a few hours or days following spontaneous vertigo (39, 40). This positional vertigo differs from “benign paroxysmal positional vertigo (BPPV)” with respect to the duration of each attack (often seconds only in BPPV vs. as long as head position is maintained in VM), duration of episodes (weeks in BPPV vs. minutes to days in VM) and presence of “nystagmus” which depends on the head position but does not match a particular semicircular canal in the case of VM (31, 41).

The most suggestive feature of VM is visual vertigo, defined as “vertigo worsened by any visual stimulation such as scrolling patterns, moving objects, and the movement of large crowds” (8, 42, 43). Like other vestibular disorders, along with vertigo, patients with VM may have phonophobia (intolerance to loud sound), osmophobia (psychological hypersensitivity to odors), photophobia (abnormal intolerance to visual perception of light), and visual or other auras (31). An aura is a temporary important neurological phenomenon that appears before or during a headache (44). The duration of an aura can differ from a few seconds to several minutes or several hours and sometime to a few days (5, 13, 18, 45, 46); however, the diagnostic criteria of VM require an aura with a minimum duration of 5 min and the aura seldom exceed 72 h.

Furthermore, the episode of vertigo and headache may not occur together, which make more difficult to diagnose this disorder (13, 18, 28). Individuals with VM generally have lower quality of life measures (3) including depression and sleep problems (47). The neurological examination of the patients with VM is usually normal between the attacks (48). During the episodes of vertigo, the patient shows signs of nystagmus and vision problem such as sensitivity to light; suggestive of either a central or peripheral vestibular abnormality (49–51).

## Treatment

Various treatment options are available for patients with VM, which includes reduction of triggers, pharmacotherapy, and physical therapy (48, 52). However, no specific evidence-based treatment is available, and no randomized controlled trials for the treatment of VM exist, mainly due to lack of diagnostic criteria (48). Rather, treatments are based on those for a migraine headache (48).

Abortive therapy is used to treat an acute attack of a headache or vertigo. The triptans like sumatriptan were reported to be effective when the vestibular symptoms were associated or not associated with a headache (53). A placebo-controlled study by Neuhauser et al. (54) investigated the drug zolmitriptan for VM attacks. The results were inconclusive because of small sample size limited to 10 patients only. In general, there are very few options available for abortive treatment of VM attacks.

The next strategies of treatment consist of prophylactic medications. Past studies (14, 29, 55) recommended the use of prophylactic drugs for VM as for a migraine, which includes antidepressants, β-blockers, and anticonvulsants. In contrast, a previous review of eight retrospective studies on the treatment of VM reported negligible improvement with a migraine prophylactic medication (14). Therefore, more high-quality studies are required to make firm evidence on the effect of prophylactic drugs in the treatment VM.

The use of nonpharmaceutical methods such as VR in the management of VM is also helpful (23) and in some patients, these approaches were found to be more effective than drugs (31). Some patients, especially those with persistent symptoms of attacks, may benefit from VR (23). The role of VR tends to become more important if secondary complications such as deconditioning, imbalance or visual dependence have developed (56, 57). However, it was not confirmed by any high quality randomized controlled studies. A recent study suggested underutilization of VR in the management of patients with VM, despite 56% of the patients reporting motion-induced symptoms (33). However, this was a retrospective study (level IV) of 90 people with VM; only 14 patients had rehabilitation so they suggest that rehabilitation may be under-utilized but the research of VR being effective in relieving headache and vestibular symptoms isn't conclusive.

## Outcome measures

There are varieties of outcome measures that are used to quantify the effects of VR. Some of them are as follows: Activities-specific Balance Confidence Scale (ABC), Dizziness Handicap Inventory (DHI), the Dynamic Gait Index (DGI). The ABC scale was designed to assess balance confidence in performing varieties of daily activities, for instance walking in the house, stair climbing, and walking on slippery floors. The questionnaire includes 16 items scored on a range from 0 to 100% (0 signify no confidence and 100 signifies full confidence) (58). The ABC scale was reported to be a valid scale in people with vestibular disorders (59). High scores indicate better and scores of 67 or less in community-living older adults have been associated with increased fall risk (60). The DHI is a reliable and valid self-report measure used in people with vertigo (61). The DHI is comprised of 25 items in three domains (functional, emotional, and physical) with three response levels. The possible total score ranges are 0–100 (62). Higher scores indicate more severe symptoms and have been associated with increased fall risk in people with balance and vestibular disorders (62). The DGI was designed to measure how well individuals can ambulate under various conditions such as walking, walking around and over objects, walking with head turns, waking and turning quickly, and going up and down steps (63). DGI include eight items, each scored on a 0–3 grade with a total score of 24. Scores of 19 or less indicate increased risk of falling in older people (64).

Other outcome measures such as vestibular symptom index (VSI), vestibular rehabilitation benefit questionnaire (VRBQ), and the vertigo symptom scale (VSS) were used to assess the effects of VR in patients with VM. The VSI is designed to evaluate the severity of six vestibular related symptoms (dizziness, balance, nausea, vertigo, headache and visual sensitivity) scored on a range from 0 to 10 (0 indicates no severity and 60 indicates maximum severity) (65). The VRBQ is designed to measure dizziness, motion provoked dizziness, anxiety, and quality of life changes following VR. Each subscale is scored separately on a six-point scale in which more negative scores indicate a negative impact (66). The VSS is designed to assess both the frequency of vestibular-balance symptoms and severity of autonomic-anxiety symptoms (67). The VSS has two versions called the long version (VSS-LV) and the short form (VSS-SF). The VSS-LV comprised of 34 items and VSS-SF includes 15 items (68). The VSS-LV can be used to assess the symptom frequency over the past year, while the VSS-SF was designed to assess symptom frequency over 1 month.

## Review of vestibular rehabilitation for patients with VM

### Search strategy

The Web of Science and PubMed electronic databases were searched until March 11, 2018. Other possible articles were searched manually from the citation list provided with each article. The potential literature search was carried out using the following keywords: (“migraine” OR “Headache”) AND (“Vestibular symptoms” OR “Vestibular disease” OR “Vestibular disorders”) AND (“Physical therapy” OR “Physiotherapy” OR “Rehabilitation” OR “Exercise” OR “Vestibular rehabilitation” OR “Vestibular treatment”).

### Quality assessment of included studies

A total of five included studies (23, 25, 69–71) investigated the effects of VR in the management of VM. The critical review form for quantitative studies was used to appraise quality assessment and risk of bias in the selected studies (72). This assessment tool is applicable to the study designs of interest in the current review and is included with a detailed user guideline (72). The user is suggested to give a score of 1 if criterion achieved, and a score of 0 if not achieved, or not reported (also 0). The user is also supposed to describe the study design and whether or not the design was appropriate for the study question? (e.g., level of knowledge, ethical issues, outcomes, etc.). In addition, authors should report reliability and validity of outcome measures to fulfill the criteria of using reliable and valid outcomes (72). If the reliability and validity of an outcome measure were not discussed, the criteria were treated “not addressed,” even if such evidence actually existed. The evaluation of risk of bias is based on sample/selection bias (item # 4), measurement/detection bias (item # 7 and 8), and performance bias (item # 9 and 10) (72) (Table 2).

**Table 2:** Results of the quality assessment using the Critical Review Form–Quantitative Studies (72).

**Study**	**Criteria**																	
	**1**	**2**	**3**	**4**	**5**	**6**	**7**	**8**	**9**	**10**	**11**	**12**	**13**	**14**	**15**	**Score**	**Scores (%)**	^*^Risk of bias
Whitney et al. (23)	Yes	Yes	Yes	**Yes**	No	No	**Yes**	**Yes**	**Yes**	**No**	Yes	Yes	Yes	No	Yes	11/15	73.3	**Yes**
Wrisley et al. (25)	Yes	Yes	Yes	**Yes**	No	No	**Yes**	**Yes**	**Yes**	**No**	No	Yes	Yes	No	Yes	10/15	66.7	**Yes**
Gottshall et al. (69)	Yes	Yes	Yes	**Yes**	No	No	**No**	**No**	**Yes**	**No**	Yes	Yes	Yes	No	Yes	9/15	60	**Yes**
Vitkovic et al. (70)	Yes	Yes	Yes	**Yes**	No	Yes	**Yes**	**Yes**	**Yes**	**No**	Yes	Yes	Yes	Yes	Yes	13/15	86.7	**Yes**
Sugaya et al. (71)	Yes	Yes	No	**Yes**	No	Yes	**Yes**	**Yes**	**Yes**	**Yes**	Yes	Yes	Yes	Yes	Yes	13/15	86.7	**No**

*1, Purpose; 2, Background; 3, Design; 4, Sample details; 5, Sample size justification; 6, Consent; 7, Reliability; 8, Validity; 9, Investigation details; 10, Contamination avoided; 11, Results as statistics; 12, Statistics appropriate; 13, Clinical importance; 14, Drop outs; 15, Appropriate conclusions*.

* *The evaluation of risk of bias is based on sample/selection bias (item # 4), measurement/detection bias (item # 7 and 8), and performance bias (item # 9 and 10) as shown in bold items*.

### Effects of vestibular rehabilitation in VM

Based on low quality evidence, nonpharmaceutical approaches such as preventing trigger factors, regular sleep and diet, and physical therapy have been shown to be effective in treating individuals with vestibular disorders including those with VM. VR is a therapeutic approach that often includes four varieties of exercise components to reduce impairments and functional limitations in patients with vestibular dysfunction: (1) exercises to improve gaze stability (gaze stability exercises), (2) exercises to adjust the symptoms (habituation exercises), (3) exercises to enhance gait and balance (gait and balance training), and (4) walking to improve endurance (73).

Table 2 illustrates the results of the quality assessment using the Critical Review Form–Quantitative Studies (72). One-third of the included studies (70, 71) had 80 and above percentage score on the Critical Review Form. Most of the included studies had a risk of bias (23, 25, 69, 70). None of the included studies provide the justification for the sample size. Only two included studies provide information about the dropouts (70, 71).

We reviewed five studies (23, 25, 69–71) that investigated the effectiveness of VR for migraine related vestibular symptoms (Table 3). Gottshall et al. (69) reported improvement in patients with migraine-associated dizziness (migraine-related vertigo) who received vestibular physical therapy. In addition, Whitney et al. (23) reported significant improvement in functional outcomes in vestibular physical therapy in patients with migraine headaches. In contrast, Wrisley et al. (25) reported fewer patients with a VM have shown improvement in all outcome measures as compared to other vestibular patients. However, none of these studies had untreated control groups and therefore, improvement could have been from time alone (time from onset) or because they were getting attention from the therapist or because they felt that they were doing something positive that gave them a sense of control over the situation. The authors cannot be sure that these improvements were from performing vestibular exercises.

**Table 3:** Study characteristics.

	**Sample size**	**Targeted population**	**Time since onset of vestibular symptoms at recruitment (months)**	**Average age at recruitment (years)**	**Outcome measurement**	**Component of vestibular rehabilitation**	**Design**	**Results**	**Conclusions**
Whitney et al. (23)	*N* = 39 (male 5, female 34)	Patients with migraine-related vestibulopathy (MRV) or vestibular dysfunction with a history of migraine headache	Mean (SD): 20.9 (26.7) Range: 1–120	Mean (SD): 54.2 (18.7) Range: 20–89	DHI ABC DGI	Habituation exercises, balance and gait training, general strengthening and stretching exercises.	Retrospective case series.	Outcomes improved following intervention. Mean DHI improvement was 12 points (*P* < 0.01); ABC improvement was 14 points (*p* < 0.01); DGI was 4 points (*P* < 0.01). 78% decrease in >1 fall was reported at discharge (*P* < 0.05). Mean reduction of baseline dizziness was 11 points (*P* < 0.05).	Patients with migraine headache and MRV showed improvement in physical function and self-perceived abilities following vestibular rehabilitation.
Wrisley et al. (25)	*N* = 62 (male 16, female 46)	Patients with VM Comparator: VI with no migraine	Mean (SD): 14.6 (NR) Range: NR	Mean (SD): 53.8 (17.8) Range: NR	DHI ABC DGI TUG	General strengthening and stretching exercises, canalith repositioning technique, habituation training, and balance retraining exercises.	Retrospective case series.	Percentage of patients who improved clinically significant amount on each outcome in patients with or without history of migraine are as follow: DHI (32% vs. 53%; *p* = 0.10); ABC (43% vs. 69%; *p* = 0.05); DGI (30% vs. 59%; *p* = 0.03); and TUG (42% vs. 50%; *p* = 0.67)	Patients with vestibular disorders with or without a history of migraine showed positive changes in both subjective and objective outcomes of balance following vestibular rehabilitation.
Gottshall et al. (69)	*N* = 34 (male 25, female 9)	Patients with migraine-related vestibular symptoms	More than 6 months Range: 6 months to 3 years	Mean (SD): 32 (NR) Range: 11–56	DHI ABC DGI CDP	Habituation exercises, balance retraining exercises, aerobic training, swimming, or cycling exercises.	A prospective cohorts study	Outcomes improved following intervention. Mean DHI improvement was 30 points (*P* < 0.05); ABC improvement was 25 points (*p* < 0.05); DGI was 4 points (*P* < 0.05); and CDP was 14 points (*P* < 0.05).	Patients with migraine-associated dizziness showed beneficial outcome following vestibular rehabilitation.
Vitkovic et al. (70)	*N* = 36 (male 13, female 23)	Patients with VM Comparator: VI with no migraine	Mean (SD): 139 (NR) Range: NR	Mean (SD): 46.8 (NR) Range: 28–70	DHI ABC VSI VRBQ FGA	Gaze stability, static tilt, habituation, gait and balance training.	A prospective assessor-blinded comparative study	Mean improvement of each outcome in patients with vestibular migraine or vestibular impairment groups are as follow: DHI (15.57 vs. 13.61); ABC (14.15 vs. 10.1); VSI (5.33 vs. 5.63); VRBQ (−15.72 vs. −22.09); and FGA (5 vs. 5.83).	This study has recommended the use of vestibular rehabilitation for VM patients who subjectively notice their symptoms more severely than other patients even though having comparable peripheral vestibular outcome.
Sugaya et al. (71)	*N* = 251 (male 56, female 195)	Patients with dizziness, VM, and tension type headache	Mean (SD): NR Range: NR	Mean (SD): 47.7 (18.2) Range: NR	HIT DHI HADS	The vestibulo-spinal reflexes (VSR) and vestibulo-ocular reflex (VOR) training exercise.	A prospective comparative study	Outcomes improved following intervention in the tension-type headache and VM groups. Mean improvements of each outcome in the tension-type headache or VM groups are as follow: HIT (6 vs. 7 points); DHI (20 vs. 20 points); and HADS (1.79 vs. 3.02).	Patients with VM, dizziness and tension-type headache demonstrated improvement of headache, dizziness, and psychological factors after vestibular rehabilitation.

*VM, vestibular migraine; DHI, dizziness handicap inventory; ABC, activities based confidence scale; VSI, vestibular symptom index; VRBQ, vestibular rehabilitation benefit questionnaire; FGA, Functional gait assessment; DGI, Dynamic Gait Index; TUG, Timed up and go test; HIT, Headache Impact Test; HADS, Hospital Anxiety and Depression Scale; CDP, computerized dynamic posturography; NR, Not reported*.

Whitney et al. (23) investigated the effect of physical therapy for patients with a diagnosis of vestibular dysfunction with a history of a migraine headache or migraine-related vestibulopathy (MRV) using the retrospective chart review of patients seen for VR program. A tailor-made VR was designed for each patient as per their symptoms and included the following components, as needed: habituation exercises, exercises to develop vestibular compensation, balance and gait training, general strengthening and stretching exercises, exercise to improve the use of specific sensory inputs for balance control. The results of the study reported significant improvement in all outcome measures following “vestibular rehabilitation” in patients with MRV or a migraine headache. They also studied the effects of anti-migraine medications along with physical therapy intervention in those patients. The result of the study found that patients who received anti-migraine medications in combination with physical therapy intervention showed improved outcomes (23). The major weakness of this study was the absence of any comparable group as this was a retrospective case series. It was unknown, whether the improvements in the outcomes were due to intervention or because of recovery of the symptoms.

Gottshall et al. (69) investigated the effects of a VR intervention along with preventive migraine medications in all patients (idiopathic migraine-associated dizziness, benign positional vertigo with migraine-associated dizziness, and posttraumatic closed-head injury with migraine-associated dizziness) with migraine-associated dizziness. They use specific exercises designed to reduce dizziness, improve balance, and improve general activity levels. The focus of the exercise was to provide specific stimuli to train the brain for habituation or attenuation of the dizziness response. Habituation or attenuation is defined as a reduction in response as a result of repeating a provocative movement many times, as described by Norre and DeWeerdt (74). To improve balance and coordinated muscle responses, balance retraining exercises were designed. To improve general activity levels, a daily aerobic training, swimming, or cycling was given. The results of the study reported a statistically significant improvement in the DHI, balance confidence score, DGI, and dynamic posture following VR for all patients (69). However, Gottshall et al. study (69) did not include any control group (nontreated) to compare the improvement seen in treated patients group. Additionally, all patients in this study continued their migraine preventive medication. Therefore, it is difficult to isolate the possible therapeutic effects of interventions and other general effects that might affect the result of the study.

Wrisley et al. (25) assessed the effect of VR using the retrospective chart review of 30 patients with or without a history of a migraine. Both the groups received a customized VR program for an average of 4.1 visits over an average duration of 3.3 months. The exercise program consisted of general strengthening and stretching exercises, canalith repositioning technique, habituation training, exercises to enhance vestibular compensation, and exercise to encourage the use of various sensory inputs to improve balance control. Patients were asked to complete the DHI, ABC Scale, DGI, and the Timed Up and Go Test. They reported clinically significant improvements in balance measures following physical therapy in vestibular disorders with or without a history of a migraine. Additionally, they found the greater perception of disability from dizziness in patients with a history of a migraine as compared to patients without a history of a migraine. The major weakness of this study was the absence of any randomly selected comparable group. Although there was a comparison group (people who didn't have migraine receiving physical therapy), it was not randomly selected as it was also a retrospective group. Therefore, it was not confirmed, whether the improvements in balance measures were due to VR or just recovery of the symptoms. In addition, the other problem with retrospective studies is that there can be bias in the data collection (outcome measures) and a lack of control over the treatments.

Recently, Vitkovic et al. (70) validated the use of VR in individuals with vestibular disorders with (VM+VI) or without (VI) a history of a migraine. All patients underwent clinical vestibular assessment, including caloric and cervical vestibular evoked myogenic potential testing to evaluate cochlear, audiometry, lateral semicircular canal and otolith function in each ear. To evaluate improvements in underlying vestibular function, a repeat testing was done between the 9-week and 6 months. A tailor-made home exercise program enough to aggravate moderate dizziness was designed for each patient. The exercise program consisted of gaze stability, static tilt, habituation, gait and balance training. These authors found heightened perception of symptoms in VM + VI patients as compared to vestibular impaired (VI) patients. Still, the VM + VI patients reported the same amount of benefit from physical therapy as VI patients in both objective and subjective measures (70). However, these authors suggested that VM + VI patients may require prolonged physical therapy to gain an acceptable level of symptoms equivalent to another group of patients at the end of rehabilitation. So the presence of VM did not prevent recovery but it may have slowed recovery. Additionally, the role of behavioral therapies along with physical therapy is important to encourage exercise compliance and providing more emotional support in these patients (70). Rehabilitation professionals treating patients with VM may encounter additional challenges in motivating exercise adherence and give extra emotional support (70). Specifically, consoling that improvement is being made despite having high levels of anxiety (75) and motion intolerance (76).

In a recent study (71), 251 patients with dizziness, including 28 patients with VM (Group 1), 144 patients without a headache (Group 2), and 79 patients with a tension-type headache (Group 3) participated in 5-day hospital-based VR. They divided all the patients into three groups: (1) VM group [patients with VM having a current headache and the Headache Impact Test (HIT) score ≥50], (2) tension-type headache group (patients with chronic and episodic tension-type headache and the HIT score ≥50), and (3) non-headache group (patients with VM without having a current headache and the HIT score ≤ 49). The HIT is comprised of 6 items designed to assess the impact of headaches on a patient's life. Four sub-groups have been divided for the interpretation of HIT scores: scores ≤ 49 indicate little or no impact, scores between 50 and 55 indicate some impact, scores between 56 and 59 indicate substantial impact, and scores ≥60 suggest severe impact (77). In this study, a group of 8–10 individuals were taught a 30-min VR program. The exercise program included repeated training of the vestibulo-spinal reflexes (VSR) and vestibulo-ocular reflex (VOR). The VSR training included static and dynamic exercises. Static exercises were: standing up and sitting down with eyes open and eyes closed, three times; standing with feet apart and eyes closed for 20 s; (4) standing with feet together and eyes closed for 20 s; standing in tandem with alternate foot position for 20 s; and single leg standing on the alternate foot for 20 s. Dynamic exercises were: 180° body turn to the left and right, three times; tandem gait walking for 10 m; and walking with head shaking (horizontal and vertical) for 10 m. The oculomotor and VOR exercise program included as follows: quick eye movement (horizontal and vertical), eye tracking (horizontal and vertical direction), and head shaking with gaze fixed target (horizontal, vertical, and oblique). Each head or eye movement was repeated for 20 times. The participants were evaluated using the DHI, HIT, and Hospital Anxiety and Depression Scale (HADS) at baseline, 1 and 4 months after their intervention. Patients with VM, dizziness and tension-type headache demonstrated a significantly improved headache, dizziness, and psychological factors following VR (71). However, the inclusion of heterogeneous groups of patients in this study limits the validity of results. Thus, further research is needed to justify using VR to reduce vestibular symptoms in these patients.

## Conclusions

From the current evidence, studies on the role of vestibular exercises in improving subjective complaints and balance in patients with VM are inconclusive. Although all studies demonstrated improvement, most of the studies do not have a control group and the subjects often had other diagnoses. Additionally, not all studies were preformed prospectively. Therefore, more randomized controlled studies are required to make firm evidence on the effect of VR in reducing vestibular symptoms in patients with VM.

## Author contributions

AA and SA significant contribution to the concept and design of the study. SA the corresponding author, drafted, analyzed, and completed the manuscript. AA revised the manuscript critically. Both the authors read and approved the final manuscript.

### Conflict of interest statement

The authors declare that the research was conducted in the absence of any commercial or financial relationships that could be construed as a potential conflict of interest.

## References

[1] Burch RC Loder S Loder E Smitherman TA. The prevalence and burden of migraine and severe headache in the United States: updated statistics from government health surveillance studies. Headache (2015) 55:21–34. 10.1111/head.12482 25600719

[2] Hamelsky SW Lipton RB Stewart WF. An assessment of the burden of migraine using the willingness to pay model. Cephalalgia (2005) 25:87–100. 10.1111/j.1468-2982.2005.00797.x 15658945

[3] Furman JM Balaban CD. Vestibular migraine. Ann N Y Acad Sci. (2015) 1343:90–6. 10.1111/nyas.12645 25728541

[4] Van Ombergen A Van Rompaey V Van de Heyning P Wuyts F. Vestibular migraine in an otolaryngology clinic: prevalence, associated symptoms, and prophylactic medication effectiveness. Otol Neurotol. (2015) 36:133–8. 10.1097/MAO.0000000000000596 25251304

[5] Dieterich M Obermann M Celebisoy N. Vestibular migraine: the most frequent entity of episodic vertigo. J Neurol. (2016) 263(Suppl. 1):S82–9. 10.1007/s00415-015-7905-2 27083888 PMC4833782

[6] Neuhauser H von Brevern M Radtke A Lempert T Population-based epidemiological evidence for the link between dizziness and migraine. In: 25th, Barany Society Meeting. Kyoto (2008):177.

[7] Szirmani A Vestibular disorders in patients with migraine. Eur Arch Otorhinolaryngol. (1997) 254(Suppl. 1):S55–7. 10.1007/BF02439724 9065628

[8] Cass SP FJ Ankerstjerne JK Balaban C Yetiser S Aydogan B. Migraine-related vestibulopathy. Ann Otol Rhinol Laryngol. (1997) 106:182–9. 10.1177/000348949710600302 9078929

[9] Parker W. Migraine and the vestibular system in adults. Am J Otol. (1991) 12:25–34. 2012186

[10] Harker LA Rassekh C. Migraine equivalent as a cause of episodic vertigo. Laryngoscope (1988) 98:160–4. 3339925 10.1288/00005537-198802000-00008

[11] Jacob RG Woody SR Clark DB Lilienfeld SO Hirsch BE Kucera GD Discomfort with space and motion: a possible marker for vestibular dysfunction assessed by a situational characteristics questionnaire. J Psychopathol Behav Assesss. (1993) 15:299–324. 10.1007/BF00965035

[12] Baloh RW. Neurotology of migraine. Headache (1997) 37:615–21. 10.1046/j.1526-4610.1997.3710615.x 9439080

[13] Cutrer FM Baloh RW. Migraine-associated dizziness. Headache (1992) 32:300–4. 1399552 10.1111/j.1526-4610.1992.hed3206300.x

[14] Fotuhi M Glaun B Quan SY Sofare T. Vestibular migraine: a critical review of treatment trials. J Neurol. (2009) 256:711–6. 10.1007/s00415-009-5050-5 19252785

[15] Radtke A von Brevern M Neuhauser H Hottenrott T Lempert T. Vestibular migraine: long-term follow-up of clinical symptoms and vestibulo-cochlear findings. Neurology (2012) 79:1607–14. 10.1212/WNL.0b013e31826e264f 23019266

[16] Jafarzadeh S Pourbakht A Bahrami E Jalaie S Bayat A. Effect of early vestibular rehabilitation on vertigo and unsteadiness in patients with acute and sub-acute head trauma. Iran J Otorhinolaryngol. (2018) 30:85–90. Available online at: http://ijorl.mums.ac.ir/article_10392_4daaec66a2de95a2de33a636175b32d6.pdf 29594074 PMC5866486

[17] Herdman SJ Hall CD Schubert MC Das VE Tusa RJ. Recovery of dynamic visual acuity in bilateral vestibular hypofunction. Arch Otolaryngol Head Neck Surg. (2007) 133:383–9. 10.1001/archotol.133.4.383 17438254

[18] Johnson GD. Medical management of migraine-related dizziness and vertigo. Laryngoscope (1998) 108(1 Pt 2):1–28. 10.1097/00005537-199801001-00001 9430502

[19] Shepard NT Telian SA Smith-Wheelock M. Habituation and balance retraining therapy. A retrospective review. Neurol Clin. (1990) 8:459–75. 2359384

[20] Cowand JL Wrisley DM Walker ML Strasnick B Jacobson JT. Efficacy of vestibular rehabilitation. Otolaryngol Head Neck Surg. (1998) 118:49–54. 10.1016/S0194-5998(98)70374-2 9450828

[21] Szturm T Ireland DJ Lessing-Turner M. Comparison of different exercise programs in the rehabilitation of patients with chronic peripheral vestibular dysfunction. J Vestib Res. (1994) 4:461–79. 7850042

[22] Brown KE Whitney SL Marchetti GF Wrisley DM Furman JM. Physical therapy for central vestibular dysfunction. Arch Phys Med Rehabil. (2006) 87:76–81. 10.1016/j.apmr.2005.08.003 16401442

[23] Whitney SL Wrisley DM Brown KE Furman JM. Physical therapy for migraine-related vestibulopathy and vestibular dysfunction with history of migraine. Laryngoscope (2000) 110:1528–34. 10.1097/00005537-200009000-00022 10983955

[24] Suarez H Arocena M Suarez A De Artagaveytia TA Muse P Gil J. Changes in postural control parameters after vestibular rehabilitation in patients with central vestibular disorders. Acta Otolaryngol. (2003) 123:143–7. 10.1080/0036554021000028109 12701729

[25] Wrisley DM Whitney SL Furman JM. Vestibular rehabilitation outcomes in patients with a history of migraine. Otol Neurotol. (2002) 23:483–7. 10.1097/00129492-200207000-00016 12170150

[26] Herdman SJ Clendaniel RA Mattox DE Holliday MJ Niparko JK. Vestibular adaptation exercises and recovery: acute stage after acoustic neuroma resection. Otolaryngol Head Neck Surg. (1995) 113:77–87. 10.1016/S0194-5998(95)70148-6 7603726

[27] Bisdorf A von Brevern M Lempert T Newman-Toker DE Classification of vestibular symptoms: towards an international classifcation of vestibular disorders. J Vestib Res. (2009) 19:1–13. 10.3233/VES-2009-0343 19893191

[28] Brandt T Dieterich M. The dizzy patient: don't forget disorders of the central vestibular system. Nat Rev Neurol. (2017) 13:352–62. 10.1038/nrneurol.2017.58 28429801

[29] Neuhauser H Leopold M von Brevern M Arnold G Lempert T. The interrelations of migraine vertigo and migrainous vertigo. Neurology (2001) 56:436–41. 10.1212/WNL.56.4.436 11222783

[30] Cherchi M Hain TC. Migraine-associated vertigo. Otolaryngol Clin North Am. (2011) 44:367–75.viii–ix. 10.1016/j.otc.2011.01.008 21474011

[31] Neuhauser H Lempert T. Vestibular migraine. Neurol Clin. (2009) 27:379–91. 10.1016/j.ncl.2008.11.004 19289221

[32] Neuhauser H Lempert T. Vertigo and dizziness related to migraine: a diagnostic challenge. Cephalalgia (2004) 24:83–91. 10.1111/j.1468-2982.2004.00662.x 14728703

[33] Power L Shute W McOwan B Murray K Szmulewicz D. Clinical characteristics and treatment choice in vestibular migraine. J Clin Neurosci. (2018) 52:50–3. 10.1016/j.jocn.2018.02.020 29550250

[34] von Brevern M Neuhauser H. Epidemiological evidence for a link between vertigo and migraine. J Vestib Res. (2011) 21:299–304. 10.3233/VES-2011-0423 22348934

[35] Bisdorff A Andree C Vaillant M Sandor PS. Headache-associated dizziness in a headache population: prevalence and impact. Cephalalgia (2010) 30:815–20. 10.1177/0333102409353617 20647172

[36] Vukovic V Plavec D Galinovic I Lovrencic-Huzjan A Budisic M Demarin V. Prevalence of vertigo, dizziness, and migrainous vertigo in patients with migraine. Headache (2007) 47:1427–35. 10.1111/j.1526-4610.2007.00939.x 18052952

[37] Headache Classification Committee of the International Headache Society (IHS) The International Classification of Headache Disorders, 3rd Edition (beta version). Cephalalgia (2013) 33:629–808. 10.1177/0333102413485658 23771276

[38] Lempert T Olesen J Furman J Waterston J Seemungal B Carey J. Vestibular migraine: diagnostic criteria. J Vestib Res. (2012) 22:167–72. 10.3233/VES-2012-0453 23142830

[39] Moretti G Manzoni GC Caffarra P Parma M. “Benign recurrent vertigo” and its connection with migraine. Headache (1980) 20:344–6. 10.1111/j.1526-4610.1980.hed2006344.x 7216756

[40] Slater R. Benign recurrent vertigo. J Neurol Neurosurg Psychiatry (1979) 42:363–7. 10.1136/jnnp.42.4.363 458483 PMC490208

[41] Kuritzky A Ziegler DK Hassanein R. Vertigo, motion sickness and migraine. Headache (1981) 21:227–31. 10.1111/j.1526-4610.1981.hed2105227.x 7287423

[42] Waterston J. Chronic migrainous vertigo. J Clin Neurosci. (2004) 11:384–8. 10.1016/j.jocn.2003.08.008 15080953

[43] Drummond PD. Triggers of motion sickness in migraine sufferers. Headache (2005) 45:653–6. 10.1111/j.1526-4610.2005.05132.x 15953297

[44] Aminoff RP Simon DA Greenberg MJ. Clinical Neurology. 7th ed. New York, NY: Lange Medical Books/McGraw-Hill (2009).

[45] Tintinalli JE Emergency Medicine: A Comprehensive Study Guide (Emergency Medicine (Tintinalli)). New York, NY: McGraw-Hill Companies (2010).

[46] Versino M Sances G Anghileri E Colnaghi S Albizzati C Bono G. Dizziness and migraine: a causal relationship? Funct Neurol. (2003) 18:97–101. Available online at: https://www.functionalneurology.com/common/php/portiere.php?ID=87a1a44ccf371d3c07d12f5f5d0ae9e5 12911141

[47] Salhofer S Lieba-Samal D Freydl E Bartl S Wiest G Wober C Migraine and vertigo–a prospective diary study. Cephalalgia (2010) 30:821–8. 10.1177/0333102409360676 20647173

[48] Furman JM Marcus DA Balaban CD. Vestibular migraine: clinical aspects and pathophysiology. Lancet Neurol. (2013) 12:706–15. 10.1016/S1474-4422(13)70107-8 23769597

[49] von Brevern M Zeise D Neuhauser H Clarke AH Lempert T (2005). Acute migrainous vertigo: clinical and oculographic findings. Brain 128(Pt 2), 365–74. 10.1093/brain/awh351 15601663

[50] von Brevern M Radtke A Clarke AH Lempert T. Migrainous vertigo presenting as episodic positional vertigo. Neurology (2004) 62:469–72. 10.1212/01.WNL.0000106949.55346.CD 14872034

[51] Polensek SH Tusa RJ. Nystagmus during attacks of vestibular migraine: an aid in diagnosis. Audiol Neurootol. (2010) 15:241–6. 10.1159/000255440 19893305

[52] Spiegel R Rust H Baumann T Friedrich H Sutter R Göldlin M. Treatment of dizziness: an interdisciplinary update. Swiss Med Wkly. (2017) 147:w14566. 10.4414/smw.2017.14566 29282702

[53] Lauritsen CG Marmura MJ. Current treatment options: vestibular migraine. Curr Treat Options Neurol. (2017) 19:38. 10.1007/s11940-017-0476-z 28965306

[54] Neuhauser H Radtke A von Brevern M Lempert T. Zolmitriptan for treatment of migrainous vertigo: a pilot randomized placebo-controlled trial. Neurology (2003) 60:882–3. 10.1212/01.WNL.0000049476.40047.A3 12629256

[55] Cha YH. Migraine-associated vertigo: diagnosis and treatment. Semin Neurol. (2010) 30:167–74. 10.1055/s-0030-1249225 20352586 PMC5682200

[56] Bisdorff AR (2011). Management of vestibular migraine. Ther Adv Neurol Disord. 4:183–91. 10.1177/1756285611401647 21694818 PMC3105632

[57] Furman JM Balaban CD Jacob RG Marcus DA. Migraine-anxiety related dizziness (MARD): a new disorder? J Neurol Neurosurg Psychiatry (2005) 76:1–8. 10.1136/jnnp.2004.048926 15607984 PMC1739317

[58] Powell LE Myers AM. The activities-specific balance confidence (ABC) scale. J Gerontol A Biol Sci Med Sci. (1995). 50A:M28–34. 7814786 10.1093/gerona/50a.1.m28

[59] Whitney SL Hudak MT Marchetti GF. The activities-specific balance confidence scale and the dizziness handicap inventory: a comparison. J Vestib Res. (1999) 9:253–9. Available online at: https://content.iospress.com/articles/journal-of-vestibular-research/ves00027 10472037

[60] Lajoie Y Girard A Guay M. Comparison of the reaction time, the Berg Scale and the ABC in non-fallers and fallers. Arch Gerontol Geriatr. (2002) 35:215–25. 10.1016/S0167-4943(02)00027-4 14764360

[61] Jacobson GP Newman CW. The development of the dizziness handicap inventory. Arch Otolaryngol Head Neck Surg. (1990) 116:424–7. 2317323 10.1001/archotol.1990.01870040046011

[62] Whitney SL Wrisley DM Brown KE Furman JM. Is perception of handicap related to functional performance in persons with vestibular dysfunction? Otol Neurotol. (2004) 25:139–43. 15021773 10.1097/00129492-200403000-00010

[63] ShumwayCook A Gruber W Baldwin M Liao S The effect of multidimensional exercises on balance, mobility, and fall risk in community-dwelling older adults. Phys Ther. (1997) 77:46–57. 10.1093/ptj/77.1.46 8996463

[64] Shumway-Cook A Brauer S Woollacott M. Predicting the probability for falls in community-dwelling older adults using the Timed Up & Go Test. Phys Ther. (2000) 80:896–903. 10.1093/ptj/80.9.896 10960937

[65] Black FO Angel CR Pesznecker SC Gianna C. Outcome analysis of individualized vestibular rehabilitation protocols. Am J Otololaryngol. (2000) 21:543–51. Available online at: https://journals.lww.com/otology-neurotology/Abstract/2000/07000/Outcome_Analysis_of_Individualized_Vestibular.15.aspx 10912701

[66] Morris AE Lutman ME Yardley L. Measuring outcome from Vestibular Rehabilitation, Part I: qualitative development of a new self-report measure. Int J Audiol. (2008) 47:169–77. 10.1080/14992020701843129 18389412

[67] Yardley L Masson E Verschuur C Haacke N Luxon L. Symptoms, anxiety and handicap in dizzy patients: development of the vertigo symptom scale. J Psychosom Res. (1992) 36:731–41. 10.1016/0022-3999(92)90131-K 1432863

[68] Duracinsky M Mosnier I Bouccara D Sterkers O Chassany O. Literature review of questionnaires assessing vertigo and dizziness, and their impact on patients' quality of life. Value Health (2007) 10:273–84. 10.1111/j.1524-4733.2007.00182.x 17645682

[69] Gottshall KR MR Hoffer ME. Vestibular rehabilitation for migraine-associated dizziness. Int Tinnitus J. (2005) 11:81–4. Available online at: http://www.tinnitusjournal.com/articles/vestibular-rehabilitation-formigraineassociated-dizziness.pdf 16419697

[70] Vitkovic J Winoto A Rance G Dowell R Paine M. Vestibular rehabilitation outcomes in patients with and without vestibular migraine. J Neurol. (2013) 260:3039–48. 10.1007/s00415-013-7116-7 24061769

[71] Sugaya N Arai M Goto F. Is the headache in patients with vestibular migraine attenuated by vestibular rehabilitation? Front Neurol. (2017) 8:124. 10.3389/fneur.2017.00124 28421034 PMC5377541

[72] Law M Stewart D Pollock N Letts L Bosch J Westmorland M Guidelines for Critical Review Form E Quantitative Studies. (1998). Available online at: http://srs-mcmaster.ca/wp-content/uploads/2015/04/Critical-Review-Form-Quantitative-Studies-English.pdf. [Accessed 28 December 2017].

[73] Hall CD Herdman SJ Whitney SL Cass SP Clendaniel RA Fife TD. Vestibular rehabilitation for peripheral vestibular hypofunction: an evidence-based clinical practice guideline: from the american physical therapy association neurology section. J Neurol Phys Ther. (2016) 40:124–55. 10.1097/NPT.0000000000000120 26913496 PMC4795094

[74] Norre ME De Weerdt W Treatment of vertigo based on habituation. 1. Physio-pathological basis. J Laryngol Otol. (1980) 94:689–96. 7000935 10.1017/s0022215100089453

[75] Eckhardt-Henn A Best C Bense S Breuer P Diener G Tschan R. Psychiatric comorbidity in different organic vertigo syndromes. J Neurol. (2008) 255:420–28. 10.1007/s00415-008-0697-x 18338198

[76] Jeong SH Oh SY Kim HJ Koo JW Kim JS. Vestibular dysfunction in migraine: effects of associated vertigo and motion sickness. J Neurol. (2010) 257:905–12. 10.1007/s00415-009-5435-5 20041331

[77] Yang M Rendas-Baum R Varon SF Kosinski M. Validation of the headache impact test (HIT-6™) across episodic and chronic migraine. Cephalalgia (2011) 31:357–67. 10.1177/0333102410379890 20819842 PMC3057423

